# From Suicide Due to an Economic-Financial Crisis to the Management of Entrepreneurial Health: Elements of a Biographical Change Management Service and Clinical Implications

**DOI:** 10.3389/fpsyg.2019.00426

**Published:** 2019-03-04

**Authors:** Gian Piero Turchi, Antonio Iudici, Elena Faccio

**Affiliations:** Department of Philosophy, Sociology, Education and Applied Psychology, University of Padova, Padova, Italy

**Keywords:** health service, economical crisis, clinical and counseling psychology, suicide, health management and care

## Introduction

Every year, about a million people commit suicide (Department of Health and Human Services, 2000) and this is why suicide is one of the main causes of death in the world (World Health Organization, 2011). This involves the various institutions that deal with the phenomenon, both regarding public healthcare services and research on the topic. In the latter context, studies have traditionally focused on the relationship between suicide and mental disorders, focusing their attention on the nosographic aspects. The relationship between mental disorders and suicide can be historically identified, for instance in some societies of the past where suicides were considered insane or criminal, to such an extent that the assets of the whole family were confiscated as punishment (MacDonald and Murphy, 1993). Furthermore, most of the studies that make suicide coincide with a mental disorder are *post-hoc* studies, i.e., retrospective (Horwitz and Wakefield, 2007). Indeed, one of these has found that the diagnosis of mental disorder was 22% before suicide and 90% after suicide (Temoche et al., 1964). Being freed from the legacy of this juxtaposition, the suicidal phenomenon becomes an object of work that intersects multiple disciplinary fields, not excluding that of psychological or psychiatric vulnerability, but certainly it seems necessary to reconsider it in the light of the various social changes. For instance, we have seen that some historical periods and some social conditions can facilitate or contrast the numbers and the ways in which the suicide act is carried out. Indeed, there is increasing evidence that some periods of economic recession and financial difficulty end up destabilizing the identity of many people and professionals (Pompili, 2013; Pompili et al., 2014), until then considered not only mentally healthy but strong contributors to the well-being of thousands of citizens and workers. This has been reported in Greece (Madianos et al., 2011; Economou et al., 2013), in Hong Kong (Lee et al., 2010), in the Anglo-Saxon area (Gunnell et al., 1999), in the state of Minais in Brazil (Minayo, 2004), in the north-east of Italy (De Vogli et al., 2013a; Peroni and Durkheim, 2014) in Finland (Ostamo et al., 2001), in some Chinese regions (Chan and Pun, 2010), in different European regions (De Vogli et al., 2013b), and in Taiwan (Chen et al., 2010). Moreover, when we use the term “crisis” we are not just talking about an individual vulnerability but a complex framework of social changes, such as the insolvency of clients, including some structures of the public administration, the usury system present in some countries and the debt that in the meantime accumulates with the banks, which can limit from 1 day to the next the availability of credit (Eures, 2012). And at a time when some people cannot imagine a future that would allow them to safeguard the image of themselves as high contributors to the development of their community, we have been faced with dramatic news reports, including the recourse to extreme acts such as suicide. In many situations, suicide is an extreme gesture based on perceived responsibilities, particularly with regard to one's own family but also to the families of one's own employees (Bortolussi, 2012; Turchi and Laugelli, 2017). This implies the need to direct the cognitive gaze toward the interactions between people and to the contextual social and economic conditions. There is, however, a different study tradition that configures suicide as an intentional act based on an improper solution to a complex existential situation (Baechler, 1975; Nunes, 1998; Lester and Thomas, 2000; Durkheim, 2005; Van Orden et al., 2010) and is distinguished from the traditional approach according to which suicide is the effect of a mental disorder (Gunnell and Frankel, 1994; Brodsky et al., 2001; Ernst et al., 2004). In the first perspective, suicide is considered within the broader biographical pathway of the person who commits it, thus configuring it within the framework of the self-determining choices made based on one's own values. It is therefore an identity recognition process that extends between the symbolic poles of life and non-life, which affects everyone and is potentially always present.

## Method and Objective

The Opinion required acquiring some data on the few international emergency suicide services available and aimed at providing a system of conceptual and organizational elements to set up Suicide Emergency Management Services under the context of entrepreneurship, also in light of the long-term experience of the Inoltre Service. It is the first service in the world that has been specifically established for emergency management and is currently active in Italy in the Veneto region (Turchi and Cigolini, 2017). Our study concentrated on studies that referred to emergency services dealing with entrepreneurs who were undergoing a crisis and did not consider studies dealing with suicide for other categories or not relating to specific services. The works referred to are about 40, identified in Scopus and Wos, however our Opinion was based mainly on the Italian Service mentioned above, the only specific on entrepreneurs that we found in the literature.

## Results

Conceptual Guidelines for the Construction of an Emergency Service:

### The Service Must Go to the User and Not the User to the Service

Although suicide is considered in intentional terms, in many situations the choice of the act can take place in hurried and rushed moments and, tendentially, in solitude. For these reasons, any interventions and services must know how to be flexible and organize themselves to intercept such occurrences. Consequently, a 24 h availability can make it possible to intercept precisely those moments in which the act may occur almost undisturbed, for instance at night, when the person is alone and without the possibility of talking to someone. This makes it possible to offer citizens the opportunity to call when there is a need or an urgency and when they have the courage to ask for help and this could happen outside the usual working hours and outside the active hours of most services. In general terms, it is a matter of intercepting the situation at risk and creating the conditions for managing the repercussions of the economic, financial, and social crisis, helping the citizen to find an answer that allows him or her to continue to feel part of the community when, at that specific moment, he or she feels excluded from it.

### Creation of a Generative Service Network

A service dealing with crises must offer the possibility to be inserted into a network of other services extended to the entire territory, both in reference to formal centers (bodies, institutions, professionals in the legal and financial field) and informal entities (network of volunteering associations as well as completely informal aggregations), in order to collect the contribution of all professionals and build real “networks” of services revolving around the same objectives (Iudici and Maiocchi, 2014). When the situation requires the immediate activation of emergency services on the territory, the Service may contact the number 112, with which it collaborates, for the management of the emergency. Once the emergency has been dealt with, the Service can assess, in agreement with the user, a way of following up on the case, for instance through economic assistance, psychological counseling, etc. Example of an emergency situation: a citizen calls at night and, crying, declares the intention of committing suicide and to have already taken drugs at home. At this time, having the possibility to call a Service like that of Inoltre can make a difference and can create the conditions for a future change of perspective.

### Involvement of the Family and Social Network in the Interception of Emergency Situations

The Inoltre Service considers as resources to be activated not only those directly involved in the financial or economic crisis but all the people who, even indirectly, as acquaintances, citizens or workers belonging to the same company, can increase the mass of interactions that, when the suicide act is considered, is reduced to almost zero. This helps to more effectively intercept the biographical condition of the troubled entrepreneur, even when he or she considers the situation in question as helpless. In addition, the Service assists family members, friends, and acquaintances of the person in difficulty, guiding them in supporting the person and eventually convincing him or her to contact the Service. Re-creating and strengthening the network of interactions in which the entrepreneur is inserted and insertable (thus increasing the “social cohesion”), the wider community can offer its contribution by opening up new possibilities and perspectives, and giving back to the person the feeling of the importance of his or her role not only as an entrepreneur, but also as a worker, citizen, parent, in the variety of facets that enrich everyone's life.

### From the Crisis Prevention to the Management of the Biographical Emergency

In this perspective, a crisis is a moment in life when a person does not consider himself or herself able to find/identify ways to manage a problematic situation or cannot see where to ask a question or find support/help. Or it may happen that an operator of an institutional or volunteer organization is faced with a problem that he or she cannot solve or does not know whom to turn to for the most appropriate answer. At times like these, the difficulties that the person goes through can lead to a closure, or reduction, of interactions and dialogue with others who have previously been called upon Faccio (2011). This progressive isolation, together with the lack of alternative perspectives, can lead to very critical situations that can jeopardize one's own life and that of the corporate world of which the person is a member. The Service is therefore configured as a space for dialogue in which to identify together solutions that the person involved is not able to see and that can later lead to a reflection on their role and their lives. In fact, the Service aims at bringing the person back to the center in the management of the biographical crisis, involving him or her in the analysis of the situation and triggering co-participation in coping with the situation. Starting from the request made to the Service by the person in difficulty, the attempt is sharing the implications of what the person is experiencing and focusing on how he or she configures them, whether in a unique or multiple way. The intervention is aimed at opening up more possibilities in the way of configuring the problem and anchoring these possibilities to the daily life of the person in a future perspective. Therefore, asking for help from the Service is a first way to manage a person's biographical path.

### Autonomous Management of One's Biographical Path

In the management of the path, one of the objectives that can be pursued is that of learning how to manage the critical situations experienced that can be possibly occur again. Consequently, following the critical moment, the focus is on the development of skills through which the person followed by the Service can learn to manage any other “issues” that may arise in the future. The focus is on building opportunities so that the actors involved may exercise and develop specific skills, in particular the management of the uncertainty that characterizes the business activity and the prior management of any other future critical scenarios (Iudici, 2015). The Service can close its intervention in a concerted manner, on the one hand making sure that the user declares to be “out” of the emergency situation and feels able to proceed autonomously in the implementation of the next planned steps; on the other hand, the operator assesses that the user has at his or her disposal all the tools to proceed independently in the management of the situation, so that support is no longer necessary, but remains active in case of need.

### Evaluating the Development of Management Skills

With respect to each intervention, a Service such as the one described must be able to assess the effectiveness of its work. In this case, it should “measure” to what extent management skills regarding emergencies and critical situations have been generated. In this sense, it is advisable for the user to describe himself or herself at the start of the intervention and after the intervention and, at the end, to declare the satisfaction with the service received (Iudici and Gagliardo Corsi, 2017; Iudici et al., in press). This would facilitate an internal improvement of the consulting and intervention activities.

## Conclusions and Policy Recommendations

In some historical periods, events such as economic and financial crises can strongly affect the lives of citizens, their families and the companies they manage. There is a wide scientific consensus on how these aspects can destabilize the daily life and the personal and social identity of people, to the point of taking dramatic steps such as suicide or attempted suicide. Given that these situations are recognized as being cyclical (De Vogli et al., 2013b), it is the responsibility of institutions and research to identify tools and services able to anticipate and manage such situations. For this reason, an emergency management service can become an important node for intercepting suicide risk situations and for strengthening social networks, but can also represent an important opportunity for the improvement of social cohesion (Turchi and Romanelli, 2013).

## Author Contributions

GT, AI, and EF critical review of literature, discussion of proposed ideas, schedule the rationale for opinion, re-reading the text and editing, final approval to the version to be published.

### Conflict of Interest Statement

The authors declare that the research was conducted in the absence of any commercial or financial relationships that could be construed as a potential conflict of interest.
